# The association of intimate partner violence with postpartum depression in women during their first month period of giving delivery in health centers at Dessie town, 2019

**DOI:** 10.1186/s12991-020-00310-6

**Published:** 2020-10-06

**Authors:** Mogesie Necho, Asmare Belete, Yosef Zenebe

**Affiliations:** Department of Psychiatry, College of Medicine and Health Sciences, Wollo University, Dessie, Ethiopia

**Keywords:** Women, Depression, Post-delivery

## Abstract

**Background:**

Postpartum depression is a common psychiatric complaint of women following delivery and a multitude of psychosocial, maternal, newborn and husband-related factors were contributing to it. This condition has a detrimental impact on the mother–infant caregiving relationship and hastens the infant’s cognitive, emotional and social development. However, a shortage of empirical evidence existed especially in developing countries including Ethiopia. Therefore, we implemented this study to determine the magnitude of postpartum depression and its correlates.

**Methods:**

A cross-sectional survey was implemented on 378 postnatal women in the maternal and child health clinic of Dessie health centers within 4 weeks of their delivery. Postpartum depression was assessed using the Edinburgh Postnatal Depression Scale (EPDS). Intimate partner violence was operationalized as a psychological, physical and sexual abusive action imposed on women by their associates. We estimated the crude and adjusted odds ratio with its 95% CI using binary logistic regression to know the association and statistical significance was declared using a *p*-value < 0.05.

**Results:**

More than one-fourth, 102 (27%) (95% CI 22.5, 31.5) of participants were obtained to have postpartum depression. Being single from socio-demographic variables (AOR = 4.9, 95% CI 1.27, 16.74), dissatisfaction with child gender (AOR = 3.1, 95% CI 1.62, 6.69), unplanned pregnancy (AOR = 2.5, 95% CI 1.76, 7.23) and depression during current pregnancy (AOR = 3.2, 95% CI 2.81, 8.91) from pregnancy and newborn-related variables, intimate partner violence; psychological (AOR = 6.5, 95% CI 1.98, 15.85), sexual and physical violence (AOR = 3.46, 95%CI 2.34, 18.55), current husbands alcoholism (AOR = 2.2, 95% CI 1.48, 5.34) from husband/partner-related variables and current substance use (AOR = 1.8, 95% CI 1.16, 3.75) were found to have a statistically significant association with postpartum depression.

**Conclusion:**

More than one-fourth of the interviewed women (27%) were found to have postpartum depression. Being single from socio-demographic variables, dissatisfaction with child gender, unplanned pregnancy, and depression during current pregnancy from pregnancy and newborn-related variables, intimate partner violence, and current husband’s alcoholism from husband/partner-related variables and current substance use were the related factors. This suggests the need for integrating postpartum depression services into the existing postnatal maternal and child health services and basing intervention geared primarily to the mentioned factors above.

## Background

Depression in the postpartum period can be explained by symptoms of changes in sleep, sadness, and eating patterns, guilty feeling, crying, fatigue, and anxiety which are linked to the skill to care for the infant [1]. Being an important psychiatric complaint of women of reproductive age [2], postpartum depression is a principal contributor to the burden of disease among women of childbearing age within 4 weeks of giving a delivery [3]. The World Health Organization (WHO) specified that 20–40% of women in unindustrialized nations sustained antepartum and postpartum depression at a given time [4]. Specifically, 10–20% of mothers worldwide have been affected by postpartum depression [5]. Besides, it affects 19% of women in low and middle-income countries during the first month of giving a delivery [2, 6]. Moreover, one in 20 women in Ethiopia has been impacted by this clinical condition [7].

The prevalence of postpartum depression in women across continents varies widely and with time. A meta-analytic study that incorporates 53 studies from high and middle-income countries representing 38,142 participants obtained a pooled magnitude of postpartum depression to be 19% [6]. Another systematic review and meta-analysis study in India which incorporated 38 studies and 20,043 women analyzed and obtained a pooled estimate of the prevalence of postpartum depression to be 22% [8]. Moreover, earlier studies reported that depression among women in the postpartum period was 7.2% in brazil [9], 17.6% in Portuguese [10], 61.4% in Korea [11], 27.3% in China [12], 34.8% in Iraq [13], 18.6% in Qatar [14], 31.4% in India, 15.4% at a prospective cohort study in Turk [15], 13.5% and 10.3% within 2 weeks and 8 weeks postpartum at the cohort study in Al-Dakhlyia government in Oman [16], 31.7% of postnatal women in South Africa [17], 43% in Uganda [18], and 34% in Jamaica [19], 22.1% in Ethiopia [20].

Psychosocial and demographic variables cited as a contributory factor for postpartum depression includes poor social support [21], low education level, poverty [22], and childbirth without the presence of relatives [23], earlier history of depression [24], and poor woman autonomy [25]. Also, pregnancy, newborn and birth-related factors such as prime-parity [26, 27], multiple children at home [28], multi-parity [29], unwanted or negative attitude toward pregnancy [20, 30–33], premarital pregnancy [34, 35], depression during pregnancy [29, 36], miscarriage [37], and prenatal high anxiety [31] are some of the related factors for the high magnitude of postpartum depression. Moreover, husband and partner-related factors such as intimate partner violence [20, 38, 39], alcoholism in the husband [40], low husbands educational level [28], psychiatric problems in the husband [41], and husbands unemployment [30, 42] were so far identified as associated factors.

Postpartum depression in women has a detrimental effect both for the mother as well as the baby. It affects the caregiving practices of mothers thereby to early discontinuation of breastfeeding, lesser rates of protective healthcare utilization and immunization [43]. According to Feldman et al. [44], newborns of mothers with this psychiatric condition had increased negative emotionality and high cortisol reactivity, poor social engagement, and emotional regulation. Moreover, children from such mothers would be with a high risk of interpersonal problems, cognitive impairment, and behavioral abnormalities than children of non-depressed mothers [45]. Besides, problems of mother–infant bonding such as abusive behavior towards the infant and rejection of the infant [46] as well as the labiality of emotion and suicidal ideation [47] are common phenomena in women with this clinical problem.

Although developing countries share for a large number of children globally, 90% of the world's children population, indicating the high delivery/fertility rate in the region, much is not known about the occurrence rate of postpartum depression in these regions in general and Ethiopia in particular. Moreover, neurological and mental illnesses such as postpartum depression contribute to 12.3% disability-adjusted life years (DALYs) [48], even though it is an understudied health issue.

As a result, trustworthy assessments of postpartum depression in these circumstances are required for delivering a focused intervention geared towards addressing the responsible factors and enhancing infant and pregnancy outcomes. Moreover, it will be a ground for the expansion of nationwide and worldwide health plans, procedures and policy [2]. Hence, we aimed and assessed the magnitude and related factors of postpartum depression at health centers in Dessie town.

## Methods and materials

### Study design and setting

This institution-based study evaluated mental postpartum depression and its associated factors in women with postnatal follow-up at health centers in Dessie town from June 3 to 30/2019. Dessie town is located in the South Wollo zone, Amhara region, North East Ethiopia, 401 km far from the capital city of Ethiopia, Addis Ababa. The town has 18 Keble and has 350,000 populations according to 2016 to 2017 south Wollo zone statistics office data. Dessie is a low temperate region with an altitude ranging from 2470 and 2550 m above sea level [49]. The town has one referral hospital established in 1954E.C and four public health centers namely Kurkur health center, Bwanbuwha health center, Hottie health center, and Segno Gebya health center.

### Study participants

All women who have postnatal care and vaccination services record within 4 weeks of their delivery in the four HCs (Kurkur health center, Bwanbuwha health center, Hottie health center and Segno Gebya health center) in Dessie town was taken as the source population while the study population comprised women who came for postnatal care and vaccination service within 4 weeks of their delivery in the four health centers (Kurkur health center, Bwanbuwha health center, Hottie health center, and Segno Gebya health center) during the study period. Women age 15–49 years on postnatal follow-up care within 4 weeks of delivery and who were willing to accept consent to participate in the study were invited to participate whereas women who were seriously sick due to complications of delivery and above 4 weeks of the postnatal period were not recruited into the study.

The required sample size was determined with a single population proportion formula and obtained to be 378. The considerations during the determination of sample size include; prevalence of postpartum depression 33.8% from a prior study in Mizan-tepi, Ethiopia [50], a 5% margin of error and a level of confidence of 95% and 10% non-response contingency.

Systematic sampling had been applied to recruit participant women from the four health centers. The numbers of women included in the study from individual health centers were determined with proportion allocation based on earlier 3 months follow-up numbers from the registration book. To identify the sampling interval, the average number of women expected to attend postnatal visits in each health center during the study month was divided by the proportionally allocated sample size in each health center and was 2 in all of the health centers. The first woman was nominated by lottery method and then every 2 women (which are calculated for each health center) visiting the health center were nominated for the study.

### Assessment of postpartum depression and its associated factors

Data were gathered with a pre-tested and interviewer-administered structured questionnaire in the Amharic language. The questionnaire had seven parts. The first part was a questionnaire for the evaluation of socio-demographic variables.

The next section was the Edinburgh postnatal depression scale (EPDS), a questionnaire for the assessment of postpartum depression. The EPDS was developed for screening postpartum depression in the past 7 days in women at outpatient, home visiting settings, or at the 6–8-week postpartum period. The EPDS consists of 10 questions. This question can be filled within five minutes. We can score the response of the participants based on this tool as 0, 1, 2, or 3 regarding the severity of the symptom. Items marked with an asterisk (*) in the questionnaire are reverse scored (i.e., 3, 2, 1, and 0). The total score is the sum of the score of each of the 10 items. The EPDS is only a screening tool, does not diagnose depression. A score of ≥ 13 on this scale was considered as having postpartum depression [51]. The Amharic version of this tool was validated in Ethiopia and had been found to have a sensitivity, specificity and Cronbach's alpha of 78.9%, 75.3%, and 0.71%, respectively, to detect postnatal depression [52]. The third section consisted of a questionnaire for the assessment of pregnancy, newborn and birth-related variables (gravidity, parity, miscarriage and stillbirths, pregnancy-related problem, mode of delivery, pregnancy status, newborn gender, desired gender of the newborn, satisfaction with the newborn gender, weight of baby at birth, gestational age of baby at birth, baby disease/defect, current baby feeding practice, and depression during pregnancy).

The fourth part was also a questionnaire for evaluation of husband/partner-related variables (relationship with a partner, husband education, husband occupational status, help, and support from husband, current alcoholism in the husband, and psychiatric problem in the husband, intimate partner violence and family history of mental illness). The fifth section comprised questionnaires for social support of women. The Oslo 3-item scale was used in assessing women’s level of social support. A score of 9 and above on this scale was considered as good social support, whereas a score of less than 8 indicates poor social support [53].

The sixth section consisted of questions for intimate partner violence (IPV) evaluation which is consisted of physical, sexual, or psychological violence elements [54]. IPV was assessed with the questions such as physical violence as physical aggression or use of objects or weapons to produce injuries; psychological violence as threatening behavior, humiliation and insults; and sexual violence as sexual intercourse imposed using physical force or threats and imposition of acts that were considered humiliating. IPV was considered positive, if the woman answered “yes” to at least one of the questions that comprised each type of violence. The final section of the questionnaire was an evaluation tool for substance-related variables (alcohol, chat, and cigarette). Substance use was assessed whether the woman has history of use of the specified substance in a lifetime and within the last 3 months [55]. If the answer was yes in both cases, it was taken as having a substance use.

Data were collected by four BSC psychiatry nurses who were trained for 3 days. Two MSc holders in integrated clinical and community mental health professionals thoroughly and seriously follow the data collection procedures and were giving timely responses for quality impairments. Moreover, a pre-test was implemented on 19 postnatal women who visited the Dessie referral hospital 4 days before the actual period of data collection and its result had not been incorporated in the final result.

### Data management and analysis methods

After the appropriate data were collected with the mentioned instruments above, it was checked for its completeness and content. The next task performed was entering the collected data to Epi-info version-7 software and the come in data was verified for omitted value and then transferred to SPSS version 20 software package for analysis. Furthermore, we described postpartum depression and different associated variables with mean, percentages, frequency and standard deviation. Crude and adjusted odds ratio (OR) with their 95% confidence interval (CI) was employed to assess the relationship strength of postpartum depression with its independent variables. All explanatory variables with a *p*-value < 0.2 in bivariate logistic regression had been together fitted into a multivariable logistic regression model to know the independent association of the variables with postpartum depression. A *p*-value < 0.05 in the multivariable binary regression model was considered statistically significant. Hosmer and Lemeshow goodness of fit test was performed and the model was adequate.

### Ethical considerations

This study was implemented after receiving an ethical clearance from the institutional ethical committee of Wollo University that reviews ethical aspects of research works. An accompanying letter was written from Wollo University Psychiatry department to each of the health centers to get permission for data collection. Finally, a permission letter from each health centers administration was received. Data collectors explained the significance of the study to all of the participants and written consent was gained from participants before the data collection. In addition parental consent was taken for minor women. The confidentiality of the collected data was guaranteed by keeping anonymous procedures.

## Results

### Socio-demographic characteristics of the participants

The mean age of the women interviewed was 29.85 years with a standard deviation of 6.39 years and 195 (51.6%) were in the age group of 25–34 years. Two hundred eighty-three (74.9%), 162 (42.9%) and 234 (61.9%) of the interviewed women were married, have education attained diploma and above and Muslim by religion, respectively. Regarding the average monthly income of the family, 167 (44.2%) had a USD of 89.4$–178.6$ per month and the majority of interviewed women, 315 (83.3) has a good support level from society (Table 1).

**Table 1:** Socio-demographic characteristics of women in postnatal period among health centers of Dessie town, Ethiopia 2019

Variable	Category	Frequency	Percentage
Age	15–24	81	21.4
	25–34	195	51.6
	≥ 35	102	27.0
Religion	Muslim	234	61.9
	Orthodox	118	31.2
	Catholic/protestant	26	6.8
Marital status	Married and live with husband	283	74.9
	Single	49	13
	Divorced/widowed/separated	46	12.1
Educational status	No formal education	11	2.9
	Primary education	65	17.2
	Secondary education	140	37
	Diploma and above	162	42.9
Occupational status	Government employee	98	25.9
	Private employed	74	19.6
	Unemployed and housewife	179	47.4
	Student	27	7.1
Average monthly income (USD) of family	< 35.7$	8	2.1
	35.7$–89.3$	68	18
	89.4$–178.6$	167	44.2
	> 178.6$	135	35.7
Social support	Poor	63	16.7
	Good	315	83.3

1$ = 28ETB, the exchange amount of Ethiopian birr to the USA dollar at the time of data collection

### Pregnancy, newborn and birth-related factors

One hundred twenty-four (32.8%) of women were primiparous and the majority, 273 (72.2%) were delivered their recent birth with spontaneous vaginal delivery. Concerning planning to pregnancy, 248 (65.6%) have a plan for their pregnancy and the remaining 130 (34.4%) have no such a plan. Two hundred three (53.7%) of women desire their baby to be female, but there was an equal proportion of sex distribution in the newborn; 189 (50%) for both male and female sexes. Besides, 58 (15.3%) were dissatisfied with their newborn gender. Eighty-seven (23%) of women had depression during their current pregnancy. Concerning gestational age of the baby at birth, birth weight and infant feeding practices, only 39 (10.8%) were delivered at < 37 weeks of gestation, 35 (9.3) were having weight < 2500 g and 23 (6.1) of infants were using formula feeding practices, respectively (Table 2).

**Table 2:** Pregnancy, newborn and birth-related factors of women in postnatal period at health centers of Dessie town

Independent variable	Category	Frequency	Percentage
Gravidity	Primigravida	121	32.0
	Multigravida	257	68.0
Parity	Primipara	124	32.8
	Multipara	254	67.2
Miscarriage and stillbirths	Yes	17	4.5
	No	361	95.5
Pregnancy-related problem	Yes	31	8.2
	No	347	91.8
Mode of delivery	Vaginal delivery	273	72.2
	Cesarean section	54	14.3
	Instrumental	51	13.5
Pregnancy status	Planned	248	65.6
	Not planned	130	34.4
New born gender	Female	189	50.0
	Male	189	50.0
Desired gender of the newborn	Female	203	53.7
	Male	175	46.3
Satisfaction with the newborn gender	Satisfied	320	84.7
	Dissatisfied	58	15.3
Gestational age of baby at birth	≥ 37 weeks	339	89.7
	< 37 weeks	39	10.3
Weight of baby at birth	< 2500 g	35	9.3
	≥ 2500 g	343	90.7
Baby disease/defect	Yes	24	6.3
	No	354	93.7
Current baby feeding practice	Breast feeding	292	77.2
	Formula feeding	23	6.1
	Both	63	16.7
Depression during pregnancy	Yes	87	23
	No	291	77

### Husband/partner-related factors

Regarding the husband and partner-related factors of women interviewed, 56 (14.8%) had poor help and support provided by their husbands. One hundred thirty-seven (36.2%) of participant mothers reported their relationship with their mother-in-law as poor. Also, 101 (26.7%) of interviewed women reported current alcoholism by their husbands. Intimate partner violence was also reported by 90 (23.8%) of participants from which the highest share was psychological violence; 72 (19%). Concerning the educational status and husband's employment, 353 (93.4%) and 373 98.7%) of women husbands were educated and employed, respectively. Only 8 (2.1%) and 7 (1.8%) of women had a family history of mental illness and psychiatric problems in their husband, respectively (Table 3).

**Table 3:** Husband/partner-related factors of women in postnatal period at health centers of Dessie town, Ethiopia 2019

Independent variable	Category	Frequency	Percentage
Relationship with partner	Poor	31	8.2
	Good	347	91.8
Help and support from husband	Poor	56	14.8
	Good	322	85.2
Relationship with mother-in-law	Poor	137	36.2
	Good	241	63.8
Current alcoholism in husband	Yes	101	26.7
	No	277	73.3
Husband education	Yes	353	93.4
	No	24	6.6
Husbands occupational status	Employed	345	91.3
	Unemployed	32	8.7
Psychiatric problem in husband	Yes	7	1.8
	No	371	98.2
Intimate partner violence	No violence	288	76.2
	Psychological	72	19
	Sexual and physical	18	4.8
Family history of mental illness	Yes	8	2.1
	No	370	97.9

### Substances use characteristics of participants of this study

Regarding substance use, 277 (60.1%) participated women had used substances ever, from those 149 (39.4%) of them used chat. Besides, 87 (23%) of the respondent mothers reported that they used substances with in the last 3 months, and 62 (16.4%) of them use chat (Table 4).

**Table 4:** Substances use characteristics among women in postnatal period at health centers of Dessie town, Ethiopia 2019

Variable	Category	Frequency	Percentage
Ever used substance	Yes	227	60.1
	No	151	39.9
Ever use of khat	Yes	149	39.4
	No	229	60.6
Ever use of alcohol	Yes	78	20.6
	No	300	79.4
Current use of substance	Yes	87	23
	No	291	77
Current use of khat	Yes	62	16.4
	No	316	83.6
Current use of alcohol	Yes	25	6.6
	No	353	93.4

### The prevalence of postpartum depression among study participants in this study

**Fig. 1:**
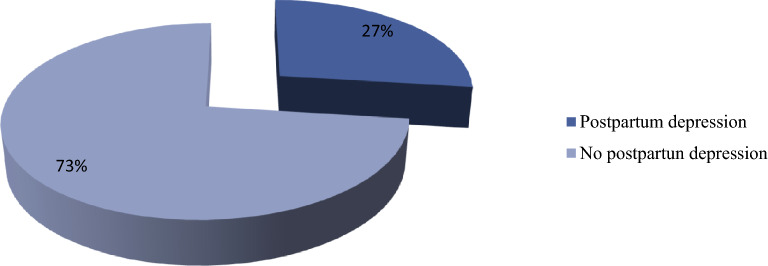
Prevalence of postpartum depression among postpartum women

We used the ten-item Edinburgh postnatal depression assessment scale to assess the outcome variable. This screening scale has 10 items which are summed together to give a total score that ranges from 0 to 30. A cut-off point ≥ 13 on this scale was used as a demarcation point between participants who have and have not postpartum depression. We, therefore, used the score ≥ 13 on this scale to identify participants with PPD and score < 13 to identify individuals who have no PPD. Based on this, 102 (27%); (95% CI 22.5, 31.5) of participants were obtained to have postpartum depression at score ≥ 13 (Fig. 1).

In this study, women who are single have a higher prevalence of PPD, 36 of 49 (73.5%) than women who were married, 60 of 283 (21.2%). Besides, the prevalence of PPD in women who have a history of intimate partner violence was much higher, 61 of 90 (67.8%) than those without partner violence, 41 of 289 (14.2%) and this difference were also statistically significant (*p*-value from Chi-square test < 0.05). Moreover, the magnitude of PPD in women delivered with vaginal, cesarean section and instrumental methods of delivery were 57/273 (20.8%), 30/54 (55.6%) and 15/51 (29.4%), respectively.

### Factors associated with postpartum depression in the study participants

In this study, being single from socio-demographic factors, dissatisfaction with one child gender, unplanned pregnancy and depression during pregnancy from pregnancy and newborn-related factors, intimate partner violence and alcoholism in the husband from husband/partner-related factors and current use of substance from substance use factors were the variables having a statistically significant association with postpartum depression in the multi-variable logistic regression with *p*-value < 0.05.

Being single is the first socio-demographic variable which has a significant association with postpartum depression. This means that single women were 5 times (AOR = 4.9, 95% CI 1.27, 16.74) more likely to develop postpartum depression than women who were married. Similarly, dissatisfaction with child gender is also an independent variable with a significant association PPD. The odds of developing PPD in women who were dissatisfied with their child's gender are three times (AOR = 3.1, 95% CI 1.62, 6.69) higher as comparatively seen with women satisfied in the child gender. Another pregnancy-related issue obtained to higher the probability of PPD in the current study is an unplanned pregnancy. Women who were pregnant but have no plan for it were 2.5 times (AOR = 2.5, 95% CI 1.76, 7.23) at the increased probability to have PPD than women who were pregnant with a plan.

The last pregnancy, newborn and child-related factor with a statistically significant association with PPD was the presence of depression during pregnancy. A participant who had depression at the time of current pregnancy was 3.2 (AOR = 3.2, 95% CI 2.81, 8.91) times at a higher odds of being depressed than those who have no history of depression during the current pregnancy. Intimate partner violence was the first husband/partner-related variable obtained to increase the odds of developing PPD in the study participants. This is to explain that women who have psychological violence were at higher odds; 6.5 times (AOR = 6.5, 95% CI 1.98, 15.85) higher probability of developing PPD than women who have no violence. Moreover, women with sexual and physical violence were 3.46 times (AOR = 3.46, 95% CI 2.34, 18.55) more probably to have PPD than women who have no intimate partner violence.

Women whose husband was drinking alcohol in the last month were also 2.2 times (AOR = 2.2, 95% CI 1.48, 5.34) at higher odds of developing PPD than women whose husband has no history of alcoholism in past 1 month. Moreover, current substance use increases the probability of developing PPD by 1.8 times (AOR = 1.8, 95% CI 1.16, 3.75) (Table 5).

**Table 5:** Bivariate and multivariable logistic regression analysis result showing the associations between the factors and postpartum depression among women having postnatal care at health centers in Dessie town, Dessie, Ethiopia, 2019

Independent variables	Postpartum depression		COR (95% CI)	AOR (95% CI)
	Yes	No		
Marital status				
Married	60	223	Reference	Reference
Single	36	13	10.3 (5.14, 20.63)	4.9 (1.27, 16.74)**
Divorced/widowed/separated	6	40	0.56 (0.23, 1.37)	0.61 (0.31, 1.20)
Satisfaction with child’s gender				
Dissatisfied	30	28	3.69 (2.07, 7.58)	3.10 (1.62, 6.69)**
Satisfied	72	248	Reference	Reference
IPV				
No violence	41	247	Reference	Reference
Psychological	50	22	13.69 (7.51, 24.37)	6.5 (1.98, 15.85)**
Sexual and physical	11	7	9.46 (3.47, 25.89)	3.46 (2.34, 18.55)**
Depression during PX				
Yes	53	34	7.69 (4.54, 13.06)	3.2 (2.81, 8.91)***
No	49	242	Reference	Reference
Unplanned pregnancy				
Yes	53	71	3.56 (2.15, 8.16)	2.5 (1.76, 7.23)*
No	43	205	Reference	Reference
Maternal current substance use				
Yes	37	50	2.57 (1.55, 4.27)	1.8 (1.16, 3.75)**
No	65	226	Reference	Reference
Social support				
Poor	10	53	0.45 (0.22, 0.94)	0.58 (0.24, 1.44)
Good	92	223	Reference	Reference
Current alcoholism in husband				
Yes	48	53	3.74 (2.29, 6.11)	2.2 (1.48, 5.34)**
No	54	223	Reference	Reference
Mode of delivery				
Vaginal	57	216	Reference	Reference
Cesarean section	30	24	4.7 (2.57, 18.72)	2.5 (0.89, 16.25)
Instrumental	15	36	1.58 (0.81, 3.01)	(0.52, 2.45)

Reference: implies groups with the lowest risk for the postpartum depression in binary logistic regression

*IPV* intimate partner violence

*p-value < 0.05, ** *p*-value < 0.01, *** *p*-value < 0.001, Hosmer and Lemeshow test (*p*-value = 0.68)

## Discussion

As per the investigator's knowledge, there is limited information regarding depression in postnatal women in Ethiopia, and no study was conducted in the study area. This scarcity of works challenges the management of women with depression in the postnatal period and health workers had different attitudes to these illnesses and on the utmost occasions underrate the problem. So, this study evaluated the rate of recurrence and related factors of postpartum depression that presented the scope of the problem and the evidence obtained would serve as baseline information to advance the health status and deliver quality services for women with the problem.

The prevalence of postpartum depression in the current study was 27% (95% CI 22.1, 31.5). With an overall magnitude of postpartum depression being 27% a minimum of 22.5% and a maximum of 31.5% of postnatal women had postpartum depression (lower and upper 95% CI of the prevalence). Being single from socio-demographic factors, dissatisfaction with child gender, unplanned pregnancy and depression during pregnancy from pregnancy and newborn-related factors, intimate partner violence and alcoholism in the husband from husband/partner-related factors and current use of substance from substance use factors were the factors associated with postpartum depression in this study.

The present study was consistent with the results of 27.3% in China [12], 31.4% in Asian [40], and 22.1% in Ethiopia [20]. However, a meta-analysis study in India which incorporated 38 studies obtained a pooled estimate of PPD to be 22% [8] and also another meta-analytic study that incorporated 53 studies obtained a pooled magnitude of PPD to be 19% [6]; both of which were lesser as compared to the current result. Moreover, studies reported PPD to be 7.2% in Brazil [9], 17.6% in Portuguese [10], 18.6% in Qatar [14], 15.4% in Turkey [15], 10.3% in Oman [16] which all are relatively lower than the present finding. The discrepancy could be due to differences in study subjects, measurement instruments, sample sizes, study designs, postnatal period timing as well as socio-cultural variances between Ethiopia and the above nations. For example, two of the above studies were meta-analytic studies so provided a more accurate pooled result that might lower the magnitude of PPD.

Besides, the study designs were a prospective cohort in Turkey [15] and Oman [16] studies. Additionally, the postnatal period considered was 8 weeks in Turkey [15] and 2 to 3 months in Portuguese study [10] that could minimize PPD magnitude. Furthermore, depression anxiety stress scale was used in Qatar [14] and the larger sample size was utilized in Qatar [14]; 1659 postnatal women and 540 women in Turkey studies [15] which all could be brought the lower prevalence of postnatal depression in these studies.

On the contrary, a study in Korea by Park et al. [11] on prevalence and risk factors for depressive symptoms in Korean women obtained that 61.4% of postnatal women had depression which was larger as compared to the result of the current study. Similarly, a study in Uganda by Kayo et al. [18] on factors associated with depressive symptoms among postpartum mothers obtained a bigger result of depression, 43%. Moreover, depression among women in the postpartum period was 31.7% in a study in South Africa by Hung et al. [17], and 34% in Jamaica by Wissart et al. [19]; both of which are larger than the present study. The smaller sample size was utilized; 155, 249, 202 and 73 postnatal women in Korea [11], South Africa [17], Uganda [18] and Jamaica [19] studies which could be brought the variation. Also, study designs were prospective cohort in Korea [11] and South Africa [17] as well as Zung self-rating depression scale was utilized to assess depression in Jamaica [19] all of which might cause the discrepancy.

Being single is the first socio-demographic variable that had a significant association with postpartum depression in the present study. This means that postnatal women who were single were 5 times more likely to develop postpartum depression than those women who were married. A study in Norway [56], Bangkok [35], India [34] and Nigeria [57] supported this association. The absence of emotional support like expressions of caring, poor informational support such as guidance and advice as well as poor instrumental support such as actual material support and task assistance from the husband in those who are single could be responsible for this association [58, 59].

Similarly, dissatisfaction with child gender is also an independent variable with a significant association with PPD. The odds of developing PPD in women who were dissatisfied with their child's gender were three times higher as comparatively seen with women satisfied in their child gender. A study in South Asia [29] that assessed 426 women for postnatal depression 5 to 10 weeks after delivery had similar findings. Another study in Thai [58] that incorporated 400 postnatal women and assessed depression 6 to 8 weeks postpartum period was also obtained a finding in line with this. However, other earlier studies defined no association of postpartum depression with dissatisfaction with the child's gender [60, 61].

Another pregnancy-related issue obtained to higher the probability of PPD in the current study was an unplanned pregnancy. Women who were pregnant but had no plans for it were 2.5 times at increased probability to have PPD than women who were pregnant with a plan. A study in Thai by Limlomwongse and Liabsuetrakulalso established that undesirable attitudes towards one's pregnancy double the risk of PPD [32]. Besides, a consistent finding was recorded with numerous earlier studies in Indonesia [30], Midwestern state [33], Turkey [31] and Ethiopia [20] in which unplanned pregnancy higher postnatal depression. Moreover, studies by Patel et al. [62] concluded that a planned pregnancy was protective from developing depression in postnatal women which further strengthen the current result. The reason for this could be the stressful nature of unplanned pregnancy [63], a higher probability of antepartum depression in women with an unplanned pregnancy [64] that might latter increases the probability of postpartum depression.

The last pregnancy, newborn and child-related factor with a statistically significant association with PPD was the presence of depression during pregnancy. The participant who had depression at the time of current pregnancy were 3.2 times at a higher odds of being depressed than those who have no history of depression during the current pregnancy. A study in Nepal [29] that assessed factors associated with depression among 426 postpartum women have a similar conclusion. Moreover, studies in Western Cheshire [24] and Turkey [36] are in agreement with such association of prior depression during pregnancy and developing depression in the postnatal period.

Intimate partner violence was the first husband/partner-related variable obtained to increase the odds of developing PPD in the study participants. This is to explain that women who have psychological violence were at higher odds; 6.5 times higher probability of developing PPD than women who have no violence. Moreover, women with sexual and physical violence were 3.46 times more probably to have PPD than women who have no intimate partner violence. A prospective cohort study among 390 postnatal women in the city of Recife [39] and a study in Ethiopia [20] supported such inference. This might be because Intimate partner violence (IPV) against women is common during pregnancy and may have adverse effects on women’s mental health during pregnancy and after delivery [65–67].

The next husband/partner-related variable which increases the probability of having PPD in women in the current study was alcoholism in the husband. Women whose husband was drinking alcohol with in the last month were 2.2 times at higher odds of developing PPD than women whose husband has no history of alcoholism in the past 1 month. A study in India [40] that assessed postpartum depression risk factors by taking a convenience sample of 58 Asian Indian immigrant women between 2 weeks and 12 months postpartum had reached a similar conclusion. Moreover, current substance use increases the probability of developing PPD by 1.8 times. This was supported by a study by Dindar and Erdogan on Turkey postpartum women [36].

### Limitations of the study

The primary limitation of the present study was that the cross-sectional design; it only designates the relationship between socio-demographic, maternal and newborn-related factors, husband and partner-related factors and substance-related factors with postpartum depression and it cannot describe the causality of such events that still requires a need for further study. The next limitation of the study was the use of EPDS to screen depression among postnatal women which is a suboptimal tool and not a diagnostic instrument; therefore over the inclusion of depressive symptoms and overestimation of depression magnitude in the postnatal women could happen.

## Conclusion

More than one in four women (27%) in the postpartum period was found to have postpartum depression in this study. Being single from socio-demographic factors, dissatisfaction with child gender, unplanned pregnancy and depression during pregnancy from pregnancy and newborn-related factors, intimate partner violence and alcoholism in the husband from husband/partner-related factors and current use of substance from substance-related factors were the variables having a statistically significant association with postpartum depression.

## Implications of the findings for clinical practice and policy

The findings of this study had important implications for clinical practitioners and policymakers. The high prevalence of postpartum depression and its association with intimate partner violence and other mentioned factors could primarily provide insight and promote intervention towards women's social and psychological health. In addition, the findings from the present study are informants of the need for raising awareness on postpartum depression and promote modifications in hospitals' and healthcare centers' policies. Above all, the finding suggests the need for integrating postpartum depression services into the existing maternal and child health services in the postpartum period. The Ethiopian federal ministry of health should incorporate postpartum depression into women health policy and should play an essential role in creating awareness of the issue to relevant stakeholders which otherwise would greatly affect the healthy mother–child parenting behavior and results in a child whose mental, emotional, cognitive and social welfare is disadvantaged. Above all, stakeholders should base their intervention geared primarily to the mentioned factors above.

## Data Availability

The data elements utilized in this research work are accessible from the corresponding author on a rational request.

## Abbreviations

AOR: Adjusted odds ratio
BSc: Bachelor of Science
CI: Confidence interval
EPDS: Edinburg Postnatal Depression Scale
HC: Health Center
IPV: Intimate partner violence
MSc: Master of Science
OR: Odds ratio
PPD: Postpartum depression
SPSS: Statistical Package for Social Sciences
